# Circadian rhythm of plasminogen activator inhibitor-1 and cardiovascular complications in type 2 diabetes

**DOI:** 10.3389/fendo.2023.1124353

**Published:** 2023-03-20

**Authors:** Yongzhuo Yu, Wenxuan Li, Lili Xu, Yangang Wang

**Affiliations:** Department of Endocrinology, The Affiliated Hospital of Qingdao University, Qingdao, China

**Keywords:** plasminogen activator inhibitor-1, circadian rhythm, cardiovascular complications, type 2 diabetes, pharmaceutical development

## Abstract

Cardiovascular complications are a common death cause in type 2 diabetes patients, as they are often combined. Plasminogen-activator Inhibitor 1 (PAI-1) participates in the development and progression of cardiovascular complications in diabetes. Insulin resistance increases PAI-1 production, and high PAI-1 levels lead to an environment conducive to thrombosis and earlier and more severe vascular disease. Current evidence also suggests that PAI-1 has a rhythmic profile of circadian fluctuations and acrophase in the morning within a single day, which might explain the high morning incidence of cardiovascular events. Thus, PAI-1 is a possible drug target. Although several PAI-1 inhibitors have been developed, none have yet been allowed for clinical use. Research on rhythm has also led to the concept of “chronotherapy”, a rhythm-based drug regimen expected to improve the treatment of cardiovascular complications in diabetic patients. Herein, we searched several databases and reviewed relevant articles to describe the circadian rhythm characteristics and endogenous molecular mechanisms of PAI-1, its relationship with insulin resistance, the causes of cardiovascular complications caused by PAI-1, and the current development of PAI-1 inhibitors. We also summarized the possibility of using the circadian rhythm of PAI-1 to treat cardiovascular complications in diabetic patients.

## 1 Introduction

Type 2 diabetes patients are often accompanied by cardiovascular disease, including atherosclerotic cardiovascular diseases (ASCVD) and heart failure (1, 2). Meanwhile, diabetes is an independent risk factor for cardiovascular disease (3–5). The risk of cardiovascular disease in diabetic patients is 2-3 times higher than that in healthy people (2, 4, 6), which is related to the thrombogenic environment and the earlier and more severe vascular pathological change (7, 8). Besides, hypofibrinolysis is a fundamental abnormality in the thrombogenic environment.

Plasminogen-activator Inhibitor 1 (PAI-1) participates in the occurrence and development of this state and is elevated in patients with type 2 diabetes, impaired glucose tolerance, and obesity. Insulin resistance and hyperglycemia, which often occur in these three states, are the main causes of elevated PAI-1 (9–11).

PAI-1 is a Serine Protease Inhibitor (SERPIN) superfamily member and shares a highly ordered structure with other members (12). PAI-1 can be produced by various cells, such as platelets, adipocytes, vascular endothelial cells, endometrial cells, and liver cells. After synthesis, PAI-1 is primarily stored in platelets, while circulating PAI-1 is mainly bound to its cofactor vitronectin, tissue-type Plasminogen Activator (t-PA), or urokinase-type Plasminogen Activator (u-PA) (13). Vitronectin is a heat-stable glycoprotein that binds to PAI-1 to stabilize its molecular conformation and can convert it to the activated form (14). PAI-1 has three molecular conformations corresponding to different functional states: active, latent, and cleaved (9). Active PAI-1 is mainly stored in platelets and is released when platelets are stimulated by thrombin, then PAI-1 rapidly binds to t-PA or u-PA, forming a stable 1:1 complex that inhibits the latter two (15). At the same time, PAI-1 can bind to fibrin and maintain inhibitory activity, contributing to clot stabilization and extension in thrombosis (16, 17). Additionally, PAI-1 is considered one of the most effective fibrinolytic modulators (18).

A series of studies have found that PAI-1 has a circadian rhythm and acrophase at around 6:30 am (Circadian rhythms were developed in evolution and fit with external time, so the time here is the geophysical time of the location where the study was conducted, and the times mentioned below follow the same) (19). Also, epidemiological evidence suggests that cardiovascular events such as myocardial infarction and aortic dissection have a morning peak (20–22). Thus, the PAI-1 rhythm might partially explain the high rate of cardiovascular events in the morning (19, 23–25), making it a possible therapeutic target (25).

The term circadian originates from the Latin *circa diem*. Circadian rhythms are present in almost all forms of life (26). In higher organisms such as humans, circadian rhythms are a complex physiological and molecular system, with the most intuitive sleep-wake cycles, feeding cycles, reward pathways, mood, and cardiovascular indicators discussed here all having circadian manifestations (27). The biological clock is a complex, genetically controlled system of molecules that controls these rhythms (28, 29). This oscillator is present in almost every organism cell, involving a transcriptional-translational feedback network and transcription factors and regulators that will be discussed in detail below.

The global incidence and prevalence of diabetes continue to increase (30), and effective and early control of cardiovascular complications, an important death cause in diabetic patients, is necessary (31). PAI-1 is a possible therapeutic target, but the research on the relationship between its circadian rhythm and diabetic cardiovascular disease is unclear, and related drugs, such as PAI-1 inhibitors, are still being developed. Therefore, based on the analysis of the characteristics and mechanisms of PAI-1 circadian rhythm generation, the elaboration of the relationship between PAI-1 and diabetic cardiovascular disease, and the assessment of the therapeutic prospects of related drugs for diabetic cardiovascular complications, we summarized the possibility of using the circadian rhythm of PAI-1 to treat the cardiovascular complications of diabetes using “chronotherapy”.

To accomplish this goal, we performed an electronic search in PubMed, Google Scholar, and SCOPUS databases to identify so far published studies using different combinations of the following keywords: plasminogen activator inhibitor-1, circadian rhythm, atherosclerotic cardiovascular diseases, cardiovascular complications, chronotherapy, drug development, type 2 diabetes mellitus. Only publications in English were considered.

## 2 PAI-1 has a circadian rhythm and is controlled by an endogenous biological clock

### 2.1 Characterization of the circadian rhythm of PAI-1

Almost all variables related to the cardiovascular system have a day/night pattern or circadian rhythms (32, 33), including the heart rate (34), blood pressure (34, 35), circulating catecholamines (36, 37), and vascular endothelial function (38). Fibrinolytic markers such as PAI-1 and tissue plasminogen activator (t-PA) also fluctuate in diurnal levels in rodents and humans (39).

Circadian fluctuations of PAI-1 have a clear correlation with activity in manifestation. Rodents-based experiments found that PAI-1 levels increased at night, the active period in rodents, and reached a minimum during the day, their resting period (40, 41). In humans, Scheer et al. conducted continuous blood sampling at 1-h intervals within one day in 12 normal people and found that PAI-1 has large amplitude (19). The acrophase was about 6:30 am, and the trough occurred around 3:30 pm. Similar rhythms were found in another trial involving 66 healthy volunteers, but the acrophase was around 8:00 am, and the nadir was 2:00 pm (24). The small number of samples in a day might limit this.

Although the characteristics of PAI-1 circadian fluctuations are related to activity and rest, the current study found that the biological clock determines PAI-1 changes during the day. Furthermore, Scheer et al. found that a mild stress test (i.e., “activity”) did not affect levels of PAI-1 (19). In addition, reversing the feeding cycle did not affect the circadian phase of circulating PAI-1 levels in mice, suggesting that the feeding cycle does not affect the circadian rhythm of PAI-1 (42). The above findings suggest that PAI-1 may have an endogenous rhythm, and feeding and activity cycles are not zeitgebers.

### 2.2 Biological basis and molecular mechanisms of the endogenous rhythm

The mechanisms of the endogenous rhythm have been previously explored. The circadian rhythms in humans and many mammals are primarily controlled by the suprachiasmatic nucleus (SCN) pacemaker neurons in the hypothalamus (43). They are mainly linked to the retina through the retinohypothalamic tract (RHT) to synchronize with ambient light rhythms (44). Pacemaker neurons isolated and cultured *in vitro* can also maintain rhythm autonomy (45).

Also, this link can still work in blinded patients. In 11 blind patients with no conscious perception of light, Czeisler et al. found that the circadian rhythm of melatonin could still be regulated by light in some patients, suggesting that the external light rhythm is an important zeitgeber (46). Furthermore, in conjunction with other studies showing that the mammalian eye is connected to at least two photosystems: the occipital cortex, which mediates the conscious perception of light and the recognition of images, and a subcortical system that mediates light-sensitive synchronization of the circadian pacemaker (46, 47).

At the cellular level, the rhythm is expressed as a diurnal variation in the spontaneous firing rate of pacemaker neurons, with a high frequency of activity during the day and a low frequency at night (48). Also, pacemaker neurons exhibit daily changes in membrane properties such as input resistance (R_input_) and resting membrane potential. This is attributed to the diurnal fluctuation of mRNA and protein levels, such as ion channels (46). Therefore, in the daytime, increased persistent sodium current (47), sodium leak current, and L-type calcium current increase excitability (49, 50). In the dark state at night, the increased potassium current makes the resting membrane potential hyperpolarize to the potassium balance potential, ensuring that neurons are not easily driven to the action potential (50, 51).

**Figure 1:**
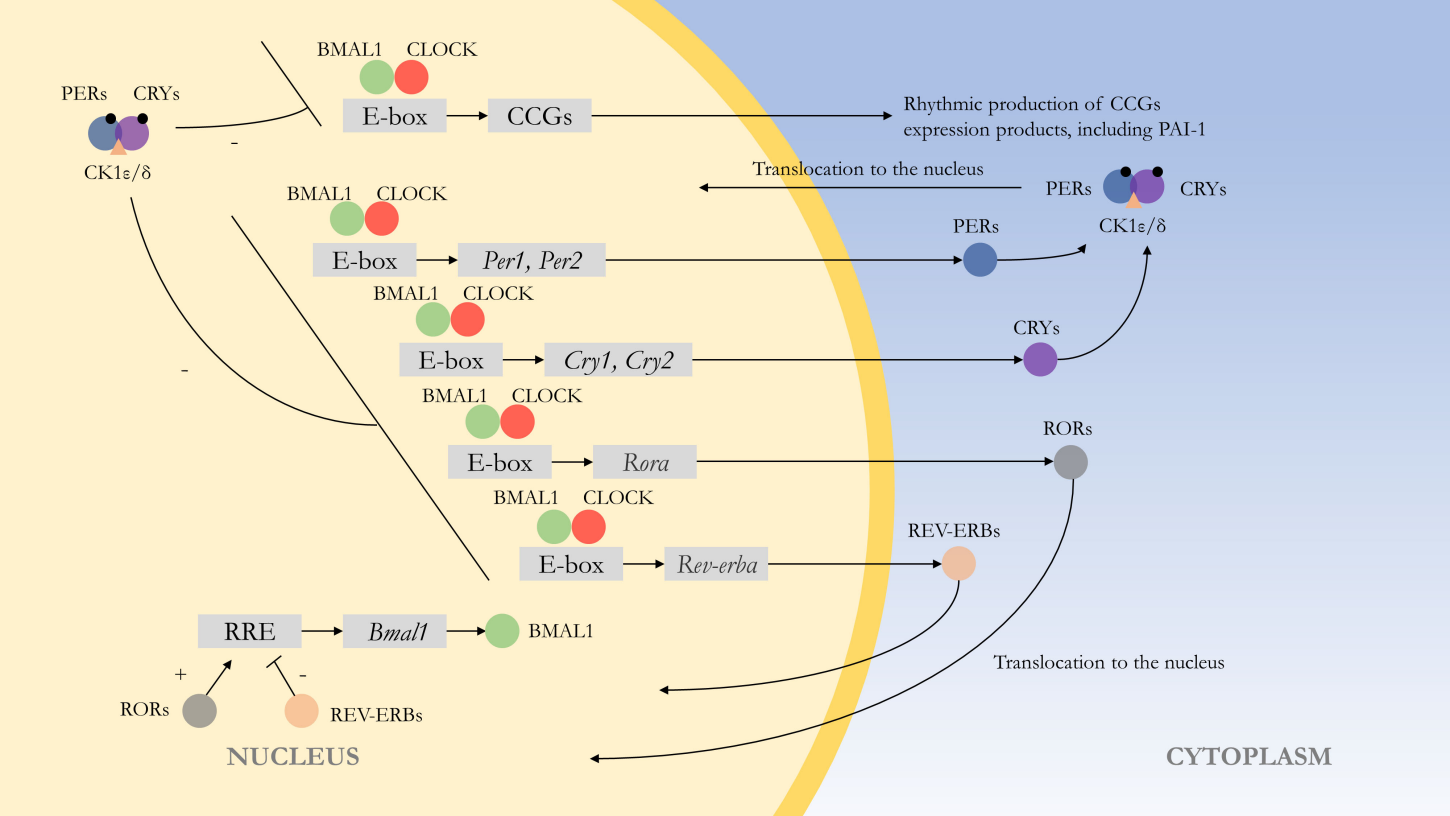
Molecular mechanism of the endogenous rhythm. The central loop is mainly composed of circadian clock genes *Clock, Bmal1, Period (Per1, Per2)*, *Cryptochrome (Cry1*, *Cry2)*, and their transcription products CLOCK : BMAL1 heterodimer and repressor proteins PER1-2 and CRY1-2. Regulation of clock control genes (CCGs) by CLOCK : BMAL1 heterodimers results in expression products with rhythmic properties, such as PAI-1. At the post-transcriptional level, PER and CRY proteins are phosphorylated, with casein kinase 1ϵ/δ (CK1ϵ/δ) and AMP kinase (AMPK) phosphorylating PER and CRY proteins, respectively, thereby enabling them to function. The CLOCK : BMAL1 heterodimer is also regulated by other feedback loops. For example, regulation by *Rev-erbα* (*Nr1d1)* and *Rorα*, with the former repressing Bmal1 transcription and the latter acting as a facilitator.(“+” represents facilitation, while “-” represents inhibition.).

At the molecular level, the rhythms are formed by autoregulatory transcriptional feedback loops of multiple genes and transcription factors (52). The central loop is mainly composed of the circadian clock genes *Clock, Bmal1, Period (Per1, Per2)*, *Cryptochrome (Cry1*, *Cry2)*, and their transcription products CLOCK : BMAL1 heterodimer and repressor proteins PER1-2 and CRY1-2. The transcription and translation products of *Clock* and *Bmal1* genes combine to form the CLOCK : BMAL1 heterodimer, which binds to the E-box of *Per* and *Cry* genes, and positively regulates the expression of the repressor proteins PER1-2 and CRY1-2. PER and CRY repressor proteins combine to translocate into the nucleus to repress their transcription (53). This feedback process lasts about 24 h and is similar to the external circadian rhythm. At the same time, CLOCK : BMAL1 regulates many downstream target genes, and most of these clock control genes (CCGs) products have diurnal fluctuations. At the post-transcriptional level, competing E3 ubiquitin ligases can degrade PER and CRY proteins, affecting their turnover rate, which can control circadian rhythms (54). Wan et al. also found that the Pai-1 gene might have an epigenetic pattern. SIRT1, a class III histone deacetylase, can bind to the *Pai-1* promoter, resulting in decreased acetylation of histone H4 lysine16 (H4K16) on this region, promoting heterochromatin formation and *Pai-1* silencing. In cell models, SIRT1 can improve vascular endothelial cell function and arterial stiffness *in vivo* and exerts a protective effect in vascular endothelial senescence (55) (**Figure 1**).

Meanwhile, SCN controls peripheral oscillators *via* nerves and hormones, and common pathways include sympathetic, parasympathetic (56), and glucocorticoids (57). Current research has demonstrated that the *Pai-1* is a CCG (39). *The Clock* gene binds directly to the promoter of PAI-1 and positively regulates its expression, which explains its circadian fluctuations (58). Using mice deficient in *Bmal1* (*Bmal1* ^−^/^−^), Somanath et al. found that knockout mice lost the circadian rhythm of PAI-1 compared to controls, shortened tail bleeding clotting time, and upregulated vWF at mRNA and protein levels (41).

Overall, the endogenous rhythm of PAI-1 is autonomous and dual. Scheer et al. suggested that this rhythm arises from evolutionary pressure (19). High PAI-1 levels at night can promote blood coagulation after injury, favoring early humans to respond to nocturnal predators. However, this rhythm increases the risk of cardiovascular events in the morning for people susceptible to cardiovascular diseases in modern society, such as type 2 diabetes patients.

## 3 Insulin resistance increases PAI-1 production, and high PAI-1 levels promote the occurrence and development of cardiovascular complications

PAI-1 levels are elevated in type 2 diabetes and obesity patients but decreased in the type 1 diabetic population. Regarding this phenomenon, Agren et al. found that insulin resistance is the leading cause of elevated PAI-1 levels (10). The combination of obesity leads to a further increase in PAI-1 levels. Thus, obese patients with type 2 diabetes show a further decrease in fibrinolytic activity (59). High blood glucose and insulin precursor molecules can also elevate PAI-1 levels (11, 60, 61). These studies suggested that type 2 diabetes can promote PAI-1 synthesis and secretion through multiple pathways.

Under normal conditions, insulin exerts its physiological effects through Protein Kinase B (PKB) activation *via* the Phosphoinositide 3-Kinase (PI3K) pathway (62). In contrast, the above signaling pathway is blocked in the insulin-resistant state. Compensatory hyperinsulinemia activates the Mitogen-Activated Protein Kinase/Extracellular signal-Regulated Kinase (MAPK/ERK) pathway (63), increasing the secretion of inflammatory markers such as Intercellular Adhesion Molecule-1 (ICAM-1), Vascular Cell Adhesion Molecule-1 (VCAM-1), and PAI-1 (64). Additionally, a study by Chen et al. on rat aortic smooth muscle revealed the presence of a cis-carbohydrate response element in the promoter region of the *Pai-1* gene and that glucose induces PAI-1 expression through two Sp1 sites in its promoter region (11). Therefore, alleviating insulin resistance and lowering blood glucose might help reduce *Pai-1* gene expression and improve hypofibrinolysis.

Insulin resistance, obesity, and lipid metabolism disorders are closely related, and the deposition of intracellular lipids can disrupt insulin signaling pathways and abnormal glucose transport and cause insulin resistance (65, 66). At the same time, insulin resistance can cause lipid disorders (67). Some studies have also found that high levels of triglyceride (TG), fatty acids, and very low-density lipoprotein (VLDL) can increase PAI-1 levels (68, 69).

Besides the already discussed reduction of fibrinolysis by PAI-1, it might also promote the formation of intimal plaques and accelerate the progression of atherosclerosis (39, 58). Thus, high PAI-1 levels have been associated with various thrombotic disorders. An intuitive manifestation of this is the higher PAI-1 levels in coronary atherosclerotic plaques in type 2 diabetic patients compared to non-diabetic patients (70), increasing the risk of disease in patients.

Furthermore, regarding the changes in the pattern of increased PAI-1 levels, Pavlov et al. analyzed the circadian rhythm of PAI-1 in acute ST-segment elevation myocardial infarction (STEMI) patients. They found that PAI-1 levels were higher than normal at all time points during the day (25). Also, Fuchs et al. found that PAI-1 levels were higher in obese patients than in healthy controls at all times and were further elevated in the presence of combined nonalcoholic fatty liver disease (71). High levels of fluctuating PAI-1 and other diurnal cardiovascular markers, combined with external stressors, increase the risk of cardiovascular events, which is mild and moderate in normal populations. However, in susceptible populations, the elevated risk might exceed the “risk threshold,” and cardiovascular events can occur (72).

In summary, in type 2 diabetes and obesity patients, multiple factors associated with insulin resistance increase PAI-1, inhibiting fibrinolysis and creating an environment of low fibrinolysis *in vivo*. High PAI-1 levels are considered an independent risk factor for thrombo-occlusive disease (73, 74). Thus, PAI-1 is involved in the development and progression of cardiovascular complications in diabetes and might explain the early and frequent onset of cardiovascular disease in type 2 diabetic patients.

## 4 PAI-1-related drugs are still in development, and several diabetes therapeutics can reduce PAI-1 levels

PAI-1 plays an essential role in the fibrinolytic pathway and is also involved in other pathophysiological processes, including cancer (75), aging (76), cardiac fibrosis (77), and wound healing (78), making PAI-1 a highly promising therapeutic target. No PAI-1-related drugs have been allowed to be used clinically, although numerous animal studies have yielded promising results.

After discovering the pharmacological potential of PAI-1, many research groups and pharmaceutical companies have been developing PAI-1 inhibitors. The drugs developed cover various types, such as monoclonal antibodies, small molecule substances, and peptides, and several review articles have been published to summarize them (9, 79, 80). Although there is a wide variety of drugs, the directions of PAI-1 inhibitors are two main categories: directly inhibit PAI-1 production and interfere with its physiological effects.

Inhibition of PAI-1 production is logically the most straightforward approach, as reduced levels represent enhanced fibrinolysis and reduced risk of thrombosis. However, reducing PAI-1 levels requires fine-tuning, and fully inhibited, or reduced levels can lead to varying degrees of bleeding (81). Various drugs (e.g., T686, T2639) have been developed to reduce PAI-1 mRNA levels, but they all exert pharmacological effects in experimental animal models (82, 83), and no clinical trial data are available. Moreover, the ideal state is to suppress the elevated PAI-1 to normal levels. Thus, the dose of the drug is difficult to control, making its clinical future relatively ambiguous.

The other mode of action, interfering with the physiological functions of PAI-1, is relatively safe and mild. For example, the mouse monoclonal anti-human PAI-1 antibody CLB-2C8 cleaves and inactivates PAI-1 and significantly enhances endogenous thrombus lysis and inhibits thrombus development in a rabbit jugular vein thrombosis model but has not been further studied in humans (84). Another antibody, MEDI-579, has high affinity and specificity for PAI-1 and modulates the interaction between PAI-1 and t-PA and u-PA (85). In the small molecule class, classical ones are PAI-039 and tiplaxtinin, and many subsequent drugs are based on improving their efficacy. PAI-039 was patented in 2004 and has shown promising results in various animal models (86). However, PAI-039 does not inhibit PAI-1 bound to vitronectin (87), which requires the development of drugs that can resist vitronectin-bound PAI-1. Although various compounds that could act on vitronectin-bound PAI-1 were subsequently developed, complete inhibition was never achieved (79), which might be related to the multiple molecular structures of PAI-1 mentioned above (**Table 1**).

**Table 1:** Overview of the development of PAI-1 related drugs.

Name of drug	Stage of the development	Date of the published data	Main findings
Drugs that directly inhibit PAI-1 production			
MPO-16R (88)	animal experiments	2001	MPO-16R is an antisense oligodeoxyribonucleotide of PAI-1 mRNA that reduces plasma PAI-1 levels in rats, and in an model of arterial thrombosis the time of vascular occlusion was significantly delayed
T686 (83)	cellular experiments	2008	T686 can inhibit the release of PAI-1 from TGF-β-stimulated cultured endothelial cells by reducing the mRNA expression
T2639 (82)	animal experiments	2010	T2639 showed good antithrombotic activity in two rat thrombosis models, with a mechanism of action similar to that of T686
Drugs that interfere with the physiological function of PAI-1			
CLB-2C8 (84)	animal experiments	1995	CLB-2C8 enhances endogenous thrombus lysis and inhibits thrombus development in a rabbit jugular vein thrombosis model
PAI-039 (86, 87)	animal experiments	2004	PAI-039 shows good inhibition of PAI-1 activity in various animal models and is considered to be a very promising drug, but PAI-039 does not inhibit PAI-1 bound to vitronectin
MEDI-579 (85)	animal experiments	2019	MEDI-579, demonstrated high affinity and specificity for PAI-1 and was able to modulate the interaction between PAI-1 and t-PA and u-PA
TM5275 (89)	animal experiments	2012	TM5275, a carboxylic acid derivatives, inherited from the previous TM5001 and TM5007, showed potent antithrombotic ability in rats and non-human primates, and was also shown to block TGF-B1-induced pulmonary fibrosis
CDE-066 (90)	animal experiments	2010	CDE-066 is a polyphenol inhibitor that effectively inhibits PAI-1 levels in PAI-1 overexpressing mice and also inhibits the physiological function of PAI-1 in the presence of vitronectin

Moreover, current research has found that drugs used to treat type 2 diabetes reduce PAI-1 levels. Recalling the role of insulin precursors mentioned previously, treatment with exogenous insulin can reduce PAI-1 levels mainly by inhibiting the secretion of insulin precursors from pancreatic β-cells (91). Insulin sensitizers such as thiazolidinediones (92) and metformin (93) can positively affect the fibrinolytic system, partly mediated by insulin resistance alleviation and PAI-1 reduction. Liu et al. found that Glucagon-like Peptide-1 receptor agonists (GLP-1RA) can inhibit PAI-1 production in human vascular endothelial cells, which might improve endothelial cell dysfunction and premature atherosclerosis in type 2 diabetes patients (94). Meanwhile, hyperglycemia can act on two Sp1 sites to promote PAI-1 production, suggesting that improved blood glucose levels are conducive to lower PAI-1 levels (11).

Additionally, various drugs for cardiovascular disease can interfere with PAI-1 levels. For example, Brown et al. evaluated the Renin-Angiotensin-Aldosterone System (RAAS) and PAI-1 and suggested that Angiotensin Converting Enzyme Inhibitors (ACEI) might also reduce PAI-1 levels (95). The TG and VLDL mentioned above can increase PAI-1 expression (68, 69), while some studies have also found that statins and Phenoxyaromatic acid lipid-regulating drugs can modulate the coagulation system and reduce PAI-1 production (96). However, some studies have also presented different views about the effects of ACEI and lipid-regulating drugs, suggesting that their effects are limited (9, 97), which might be related to the complex molecular structure of PAI-1 and its different functional states (9).

Furthermore, the thrombotic complications of severe COVID-19 disease might be directly attributable to elevated PAI-1 levels. Whyte et al. suggested that PAI-1 might serve as a significant prognostic predictor and existing drugs (e.g., tenecteplase) might be used to treat COVID-19 and other respiratory diseases (98). Also, a PAI-1 inhibitor (TM5614) has been investigated for treating patients with severe COVID-19 (Study To antagOnize Plasminogen Activator Inhibitor-1 in Severe COVID-19: Trial identifier NCT04634799, https://clinicaltrials.gov/ct2/show/NCT04634799?term=PAI-1$+$inhibitor&draw=2&rank=1). Another drug (ACT001), still in phase 1 clinical trials, is expected to inhibit the proliferation, invasion, and migration of glioblastoma in combination with cisplatin and exert anti-tumor effects (99).

In conclusion, PAI-1 is a promising therapeutic target, and multiple PAI-1 inhibitors have shown promising applications. Drugs commonly used clinically for treating type 2 diabetes can also reduce PAI-1, a phenomenon primarily associated with lower insulin precursors and improved insulin resistance, expanding the evidence for the clinical use of these drugs.

## 5 Discussion

In modern life, circadian rhythm disorders caused by unhealthy habits such as staying up late, artificial light, and night shift work are quite common. Current research has demonstrated that circadian rhythm disturbances are associated with myocardial infarction, cancer, and neurodegenerative diseases (21). Hence, circadian rhythm disorders can lead to a wide range of conditions.

From another point of view, cardiovascular events and cardiovascular indicators are affected by time, and the therapeutic effects might be most effective if the drug is administered following these rhythms. Additionally, the metabolism of drugs is also regulated by circadian rhythms (100, 101), which led to the proposition of the “chronotherapy” concept (102).

In fact, “chronotherapy” has been studied to treat cardiovascular disease. As early as 1985, Muller et al. found that beta-adrenergic receptor blockers reduced the incidence of adverse cardiovascular events and controlled their morning peak (20). Sica et al. specifically analyzed the pharmacokinetic profile of a propranolol extended-release agent and concluded that nighttime dosing is recommended to ensure morning drug concentrations (103). Besides, a prospective study followed 448 type 2 diabetic patients with comorbid hypertension for a median duration of 5.4 years and found that cardiovascular risk was significantly lower in patients who took one or more hypertensive drugs at bedtime than in subjects who took them after rising in the morning (104). These studies support “chronotherapy” effectiveness.

Therefore, PAI-1-related drugs are expected to reduce the risk of cardiovascular events if their mode of administration can be adjusted to maintain good blood concentrations at the acrophase of PAI-1 levels. However, there are no reports of PAI-1-related drugs incorporating circadian rhythms. Nevertheless, the use of chronotherapy faces some practical problems. The first obstacle is the difficulty of detecting and diagnosing circadian rhythm disorders. Detecting a complete rhythm requires continuous sampling, which must be done partly at night, making it difficult to complete in an outpatient setting and often difficult for patients to accept (33). Second, the presence of comorbidities can also affect chronotherapy effectiveness. For example, in people with comorbid obstructive sleep apnea syndrome, the peak of cardiovascular events occurs earlier in the night rather than in the morning (105). Additionally, the intersection of multiple factors makes chronotherapy limited. For example, using beta-adrenergic receptor blockers at night can reduce the risk of cardiovascular events as described above but can impair sleep by inhibiting melatonin release (106, 107).

Based on the above studies, PAI-1 has a rhythm controlled by genes, and its morning acrophase might explain the high incidence of cardiovascular events in the morning. Insulin resistance in patients with type 2 diabetes or obesity leads to an increase in PAI-1 and high levels throughout the day compared to normal individuals, creating an environment of reduced fibrinolysis that promotes the development of cardiovascular complications. Concurrently high PAI-1 levels, combined with individual susceptibility and external stressors, cardiovascular events might occur when the risk exceeds the “risk threshold” described above.

However, our current study also has some limitations. Many of the above cardiovascular indicators (e.g., heart rate, blood pressure, circulating catecholamines) have circadian rhythms, and their patterns are also associated with cardiovascular events. Some of these indicators (e.g., blood pressure) can affect PAI-1 levels (108), which makes the role of PAI-1 in this not very conclusive. Hence, further studies are needed to clarify it.

The role of PAI-1 in cardiovascular complications of diabetes has been widely studied. Although no PAI-1 inhibitors have been approved for human treatment, there is a genuine clinical need for safe and effective PAI-1-related drugs. At the same time, the discovery of the circadian clock has deepened our understanding of circadian rhythms, but it is still challenging to transpose these discoveries into clinical practice (33).

In summary, we expect a safe and effective PAI-1-associated drug with a coordinated drug use pattern that maintains good drug concentrations when PAI-1 is expected to reach its acrophase, which might reduce the risk of cardiovascular events. Finally, considering the poor prognosis of cardiovascular events, prevention, such as regular follow-up in a plain, healthy lifestyle and good glycemic control, should also be emphasized.

## Author contributions

YY contributed to the conception and the writing of the article. WL performed the framework. LX gave the constructive discussions to the article. YW revised important intellectual content critically for important intellectual content. All authors contributed to the article and approved the submitted version.
